# Biochemical data from the characterization of a new pathogenic mutation of human pyridoxine-5'-phosphate oxidase (PNPO)

**DOI:** 10.1016/j.dib.2017.10.032

**Published:** 2017-10-28

**Authors:** Martino L. di Salvo, Mario Mastrangelo, Isabel Nogués, Manuela Tolve, Alessandro Paiardini, Carla Carducci, Davide Mei, Martino Montomoli, Angela Tramonti, Renzo Guerrini, Roberto Contestabile, Vincenzo Leuzzi

**Affiliations:** a Dipartimento di Scienze Biochimiche “A. Rossi Fanelli”, Sapienza Università di Roma, Italy; b Dipartimento di Pediatria e Neuropsichiatria Infantile, Sapienza Università di Roma, Via dei Sabelli 108, 00141 Roma, Italy; c Istituto di Biologia Ambientale e Forestale, Consiglio Nazionale delle Ricerche, Monterotondo Scalo, Roma, Italy; d Dipartimento di Medicina Sperimentale, Sapienza Università di Roma, Italy; e Dipartimento di Biologia e Biotecnologie “Charles Darwin”, Sapienza Università di Roma, Italy; f Dipartimento di Neuroscienze, Azienda Ospedaliero-Universitaria Meyer, Università di Firenze, Italy; g Istituto di Biologia e Patologia Molecolari, Consiglio Nazionale delle Ricerche, Roma, Italy

**Keywords:** Pyridoxine-5′-phosphate oxidase, Epilepsy, Children, Pyridoxine

## Abstract

PNPO deficiency is responsible of severe neonatal encephalopathy, responsive to pyridoxal-5′-phosphate (PLP) or pyridoxine. Recent studies widened the phenotype of this condition and detected new genetic variants on PNPO gene, whose pathogenetic role and clinical expression remain to be established. One of these mutations, Arg116Gln, is of particular interest because of its later onset of symptoms (beyond the first months of life) and its peculiar epileptic manifestations in patients. This protein variant was expressed as recombinant protein in *E coli*, purified to homogeneity, and characterized with respect to structural and kinetic properties, stability, binding constants of cofactor flavin mononucleotide (FMN) and product (PLP) in order to define the molecular and structural bases of its pathogenicity.

For interpretation and discussion of reported data, together with the description of clinical studies, refer to the article [1] (doi: 10.1016/j.ymgme.2017.08.003).

**Specifications Table**

**Table:** 

Subject area	*Protein biochemistry*
More specific subject area	*Biochemical characterization of enzyme mutant forms*
Type of data	*Text file, Graph, Figures*
How data was acquired	*Data were acquired upon protein purification and spectrophotometric measurements. Instruments used in this work were: Spectrophotometer HP 8453 diode array, Spectropolarimeter Jasco 725, Spectrofluorimeter Horiba Jobin-Ivon FluoroMax-3, FPLC AKTA PrimePlus GE Healthcare*
Data format	*Raw, Analyzed*
Experimental factors	*Wild type and mutant PNPO proteins, purified to homogeneity prior to biochemical characterization*
Experimental features	*Catalytic constants, melting temperature, and affinity for ligands for wild type and mutant PNPO proteins were compared*
Data source location	*Rome, Italy*
Data accessibility	*Data are available with this article*

**Value of the data**

• The data describe the new purification protocol for recombinant human PNPO mutant forms.
• The data present structural and functional characterization of PNPO mutant forms.
• The data allow the determination of delivery capability of PLP from PNPO to PLP-dependent enzymes.

## 1 Data

**Fig. 1:**
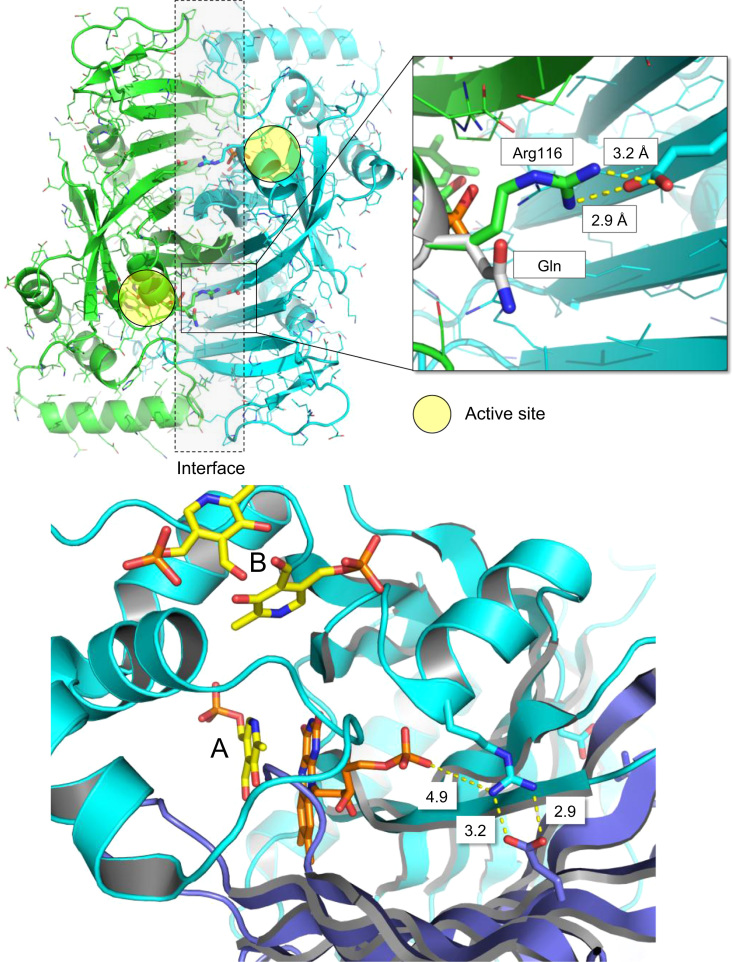
Upper panel: Structural comparison of wild type and mutant Arg116Gln form of PNPO (subunits within the dimer are shown in green and cyan). The mutation is indicated in white sticks. Distances between the atoms involved in an ion-pair interaction are also indicated, in Å. Lower panel: Close-up of the three-dimensional structure of the active site of human PNPO (PDB 1NRG; [2]. The two protein subunits are shown in cyan end blue. Secondary structures are shown as cartoon, while Arg116 and Glu143 residues are depicted as sticks. FMN and PLP in the active site (A) are shown as sticks, in orange and yellow color, respectively. Location of PLP in the secondary tight binding site (B)(shown as two different conformers) results from the superimposition with *E. coli* PNPO three-dimensional structure in the complex with PLP (PDB 1G79; [12]).

**Fig. 2:**
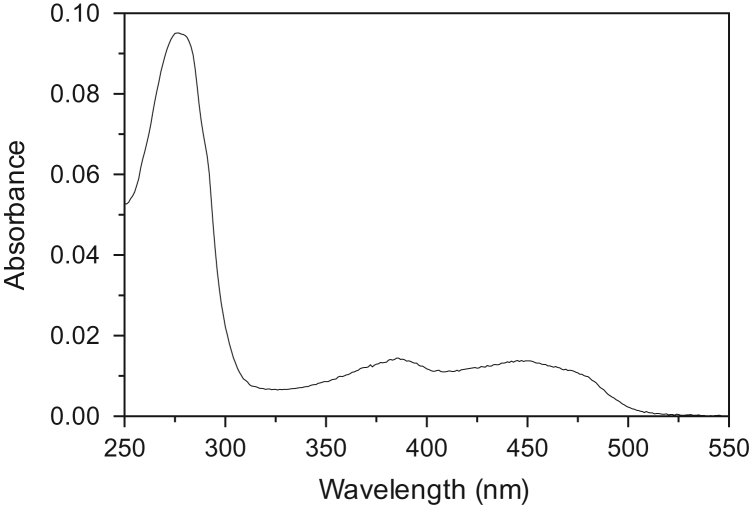
UV–Vis absorption spectrum of purified human PNPO Arg116Gln mutant form. The enzyme exhibits absorption maxima at 278 nm, 387 nm, and 450 nm typical of FMN-binding proteins.

**Fig. 3:**
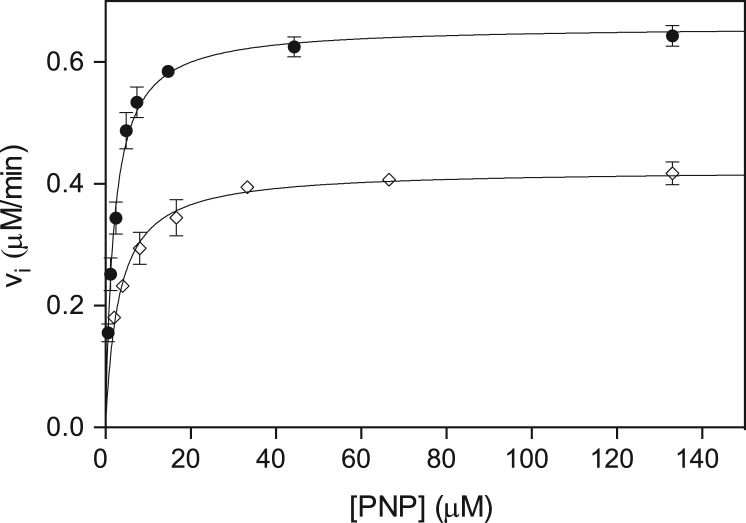
Dependence of initial velocity (v_i_) of PLP formation on PNP concentration. Wild type (closed circles, •), Arg116Gln PNPO (open squares, ◊). All experimental points are the average±standard deviation of at least three independent measurements. The continuous lines through the experimental points were obtained by non-least square fitting of experimental data to Michaelis-Menten equation.

**Fig. 4:**
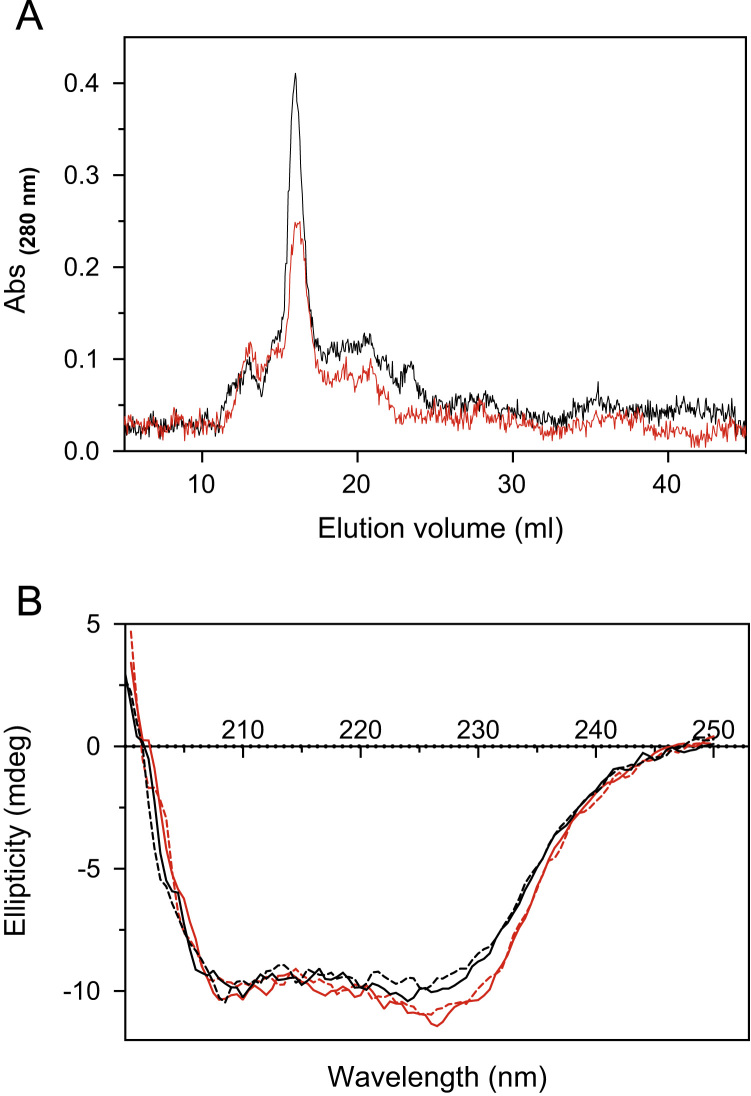
**A)** Superdex 200 10/300 FPLC size exclusion chromatography. Elution profile of wild type (black line) and Arg116Gln (red line) PNPO. Chromatographic separations were repeated using different protein batches, obtaining similar results; **B)** Far-UV CD spectra of wild type (black lines) and Arg116Gln (red lines) at 20 °C and 40 °C (continuous and dashed line, respectively).

**Fig. 5:**
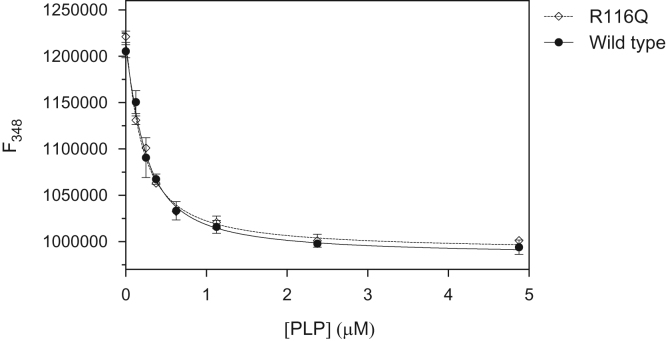
Fluorescence emission quenching of PNPO (excitation wavelength of 280 nm, emission 348 nm) upon binding to PLP: wild type (closed circles, •), Arg116Gln (open squares, ◊). The continuous (wild type) and dashed (Arg116Gln mutant) lines through the experimental points were obtained by non-least square fitting of experimental data to Eq. (1).

The data presented in this paper refer to structural and functional characterization of a new pathogenic PNPO mutant form, related to the onset of a peculiar epileptic status in patients. Fig. 1 shows insights into the active site structure of modeled human PNPO (Arg116Gln mutant form). Fig. 2, Fig. 3, Fig. 4, Fig. 5 show the characterization of Arg116Gln PNPO mutant.

## 2 Experimental design, materials and methods

### 2.1 Molecular modeling

The three-dimensional structure of human PNPO (PDB Code: 1NRG) [2] was used as a starting point to model the Arg116Gln mutant (Fig. 1). The mutation on protein structure was carried out using the “Mutate model” script [3]. Procheck [4] was used to monitor the stereochemical quality of the model, ProsaII [5] to measure the overall protein quality. Prediction of protein stability was carried out using DUET server [6].

### 2.2 Expression and purification

PNPO forms were purified following a simplified procedure derived form a previous protocol [2]. Briefly, supernatant from cell lysis was directly loaded onto a HisTrap FPLC column and eluted with imidazole (0–250 mM) in 50 mM Tris–HCl pH 7.6, 300 mM NaCl. Spectrum of Arg116Gln mutant is shown in Fig. 2. Apo-enzyme was prepared as previously described [2].

### 2.3 Kinetic studies

Enzymatic assays were performed as previously described [7]. Experimental determinations are depicted in Fig. 3. K_m_ and k_cat_ values are shown in Table 1 of Ref [1]. For enzyme stability experiments, PNPO forms were left at either 37 or 42 °C at a final enzyme concentration of 0.3 μM. Enzymatic assays were then performed with a final PNP concentration of 100 μM (See Figure 4 in Ref [1]).

### 2.4 Size-exclusion chromatography

One-hundred microliter samples of 10 μM Arg116Gln or 16 μM wild type PNPO were loaded onto a Superdex 200 FPLC column and eluted using 50 mM sodium Hepes, pH 7.6, 150 mM NaCl (Fig. 4A).

### 2.5 Circular dichroism measurements

Far-UV CD measurements were carried out at 20 and 40 °C in 50 mM sodium Hepes buffer pH 7.6, 150 mM NaCl with a protein concentration of 4 μM. The relative spectra are shown in Fig. 4B.

### 2.6 PLP binding equilibrium

Analyses took advantage of protein intrinsic fluorescence quenching observed upon PLP binding as previously described [8]. PLP binding curves are shown in Fig. 5. Data were fitted to Eq. (1), in which *F*_*rel*_ is the measured relative fluorescence at 348 nm, *F*_*0*_ is fluorescence in the absence of PLP, *F*_*inf*_ is fluorescence at infinite PLP concentration, [*PLP*] is the total PLP concentration, [*PNPO*] stands for the total protein concentration, and *K*_*d*_ is the dissociation constant of the equilibrium $\mathrm{PLP}+\mathrm{PNPO}⇌{\mathrm{PNPO}}_{\mathrm{PLP}}$, where [*PNPO*_*PLP*_] represents the concentration of PNPO•PLP complex.

(1) $$F_{\mathrm{rel}}=F_{0}-\left(F_{0}-F_{\inf}\right)\times \frac{\frac{\left[\mathrm{PLP}\right]+\left[\mathrm{PNPO}\right]+K_{d}-\sqrt{{\left(\left[\mathrm{PLP}\right]+\left[\mathrm{PNPO}\right]+K_{d}\right)}^{2}-4\left[\mathrm{PNPO}\right][\mathrm{PLP}]}}{2}}{[\mathrm{PNPO}]}$$

The fraction in Eq. (1) corresponds to the fraction of enzyme-bound PLP at equilibrium $\left(\frac{{[{\mathrm{PNPO}}_{\mathrm{PLP}}]}_{\mathrm{eq}}}{\left[\mathrm{PNPO}\right]}\right)$. ${\left[{\mathrm{PNPO}}_{\mathrm{PLP}}\right]}_{\mathrm{eq}}$ was derived from the equation for the dissociation constant of the binding equilibrium,

$$K_{d}=\frac{\left(\left[\mathrm{PNPO}\right]-\left[{\mathrm{PNPO}}_{\mathrm{PLP}}\right]\right)\times \left(\left[\mathrm{PLP}\right]-[{\mathrm{PNPO}}_{\mathrm{PLP}}]\right)}{[{\mathrm{PNPO}}_{\mathrm{PLP}}]}$$

as one of the two solutions of the quadratic equation

$${\left[{\mathrm{PNPO}}_{\mathrm{PLP}}\right]}^{2}-\left(K_{d}+\left[\mathrm{PLP}\right]+\left[\mathrm{PNPO}\right]\right)\times \left[{\mathrm{PNPO}}_{P\mathrm{LP}}\right]+\left[\mathrm{PNPO}\right]\times \left[\mathrm{PLP}\right]=0$$

### 2.7 FMN binding

Dissociation constant for FMN binding to PNPO forms were analyzed by FMN fluorescence quenching observed upon binding of the cofactor to apo-PNPO [9]. The saturation curves are shown in Figure 3A of Ref [1]. The relative K_d_ values are reported in Table 1 of Ref [1]. Data were analyzed according to Eq. (2), in which *F*_*rel*_ is the measured relative fluorescence at 525 nm, *F*_*0*_ is fluorescence in the absence of apo-PNPO, *F*_*inf*_ is fluorescence at infinite apo-PNPO concentration, [*APO*] is the total apo-enzyme concentration, [*FMN*] stands for the total cofactor concentration and *K*_*d*_ is the dissociation constant of the equilibrium APO+FMN⇌HOLO

(2) $$F_{\mathrm{rel}}=F_{0}-\left(F_{0}-F_{\inf}\right)\times \frac{\frac{\left[\mathrm{APO}\right]+\left[\mathrm{FMN}\right]+K_{d}-\sqrt{{\left(\left[\mathrm{APO}\right]+\left[\mathrm{FMN}\right]+K_{d}\right)}^{2}-4\left[\mathrm{FMN}\right][\mathrm{APO}]}}{2}}{[\mathrm{FMN}]}$$

The fraction in Eq. (2) corresponds to the fraction of enzyme-bound FMN at equilibrium $\left(\frac{{\left[\mathrm{HOLO}\right]}_{\mathrm{eq}}}{\left[\mathrm{FMN}\right]}\right)$. $\left[{\mathrm{HOLO}}_{\mathrm{eq}}\right]$ was derived from the equation for the dissociation constant of the binding equilibrium,

$$K_{d}=\frac{\left(\left[\mathrm{FMN}\right]-\left[{\mathrm{HOLO}}_{\mathrm{eq}}\right]\right)\times \left(\left[\mathrm{APO}\right]-[{\mathrm{HOLO}}_{\mathrm{eq}}]\right)}{[{\mathrm{HOLO}}_{\mathrm{eq}}]}$$

as one of the two solutions of the quadratic equation

$${\left[{\mathrm{HOLO}}_{\mathrm{eq}}\right]}^{2}-\left(K_{d}+\left[\mathrm{APO}\right]+\left[\mathrm{FMN}\right]\right)\times \left[{\mathrm{HOLO}}_{\mathrm{eq}}\right]+\left[\mathrm{FMN}\right]\times \left[\mathrm{APO}\right]=0$$

### 2.8 Thermal denaturation

Protein samples (4 μM), were heated from 20 to 90 °C monitoring the dichroic activity at 220 nm [10]. Denaturation curves were fitted to Eq. (3), in which θ_220_ is the measured ellipticity at 220 nm, Δθ is the maximum ellipticity change, *T* is the temperature expressed in Celsius, *T*_m_ is the apparent melting temperature, and *n* is the steepness of the sigmoid curve [10]:

(3) $$\theta_{220}=\left(\Delta \theta \frac{T^{n}}{T_{m}^{n}+T^{n}}\right)+\mathrm{const}$$

Denaturation curves are shown in Figure 3B of Ref [1].

### 2.9 Stoichiometry of PNPO•PLP complexes

Wild type or Arg116Gln PNPO (150 μM) were mixed with 450 μM PLP and incubated at 30 °C for one hour. Samples were run on P-6DG Bio-Gel. Fractions were analyzed by absorption spectra to detect the presence of protein and PLP. Protein concentration was measured by Bradford assay. PLP concentration was measured as in [11].

### 2.10 Transfer of tightly bound PLP

Protein samples saturated with PLP (PNPO•PLP complexes) were mixed at a final concentration of 2 µM with equimolar amount of apo-human cytosolic serine hydroxymethyltransferase (hcSHMT). At various time intervals, transfer of PLP was determined by measuring hcSHMT catalytic activity [11]. PLP transfer kinetics are shown in Figure 5 of Ref [1].
